# Jaundice and Haematemesis: An Unusual Presentation of Metastatic Malignant Melanoma

**DOI:** 10.7759/cureus.8035

**Published:** 2020-05-09

**Authors:** Philip S Rothschild, Arjun Subramaniam, Rahul Chakrabarti

**Affiliations:** 1 Medicine, The University Hospital Geelong, Geelong, AUS; 2 General Medicine, Launceston General Hospital, Launceston, AUS; 3 Ophthalmology, Royal Victorian Eye and Ear Hospital, Melbourne, AUS

**Keywords:** melanoma, jaundice, haematemesis

## Abstract

An 87-year-old male presented with jaundice and haematemesis on a background of recent lethargy and a history of excessive alcohol use. The results of a computed tomography (CT) scan indicated either a cirrhotic liver with regenerative nodules or diffuse malignancy. A gastroscopy revealed an ulcerating gastric tumour. The gastric biopsy confirmed the neoplasm as metastatic malignant melanoma, and the patient passed away on the day of diagnosis from acute hepatic failure. This case is unusual as there was an atypical cause of jaundice and haematemesis, and the diagnosis of melanoma was not established until the day of the patient’s death.

## Introduction

When most doctors hear of a patient presenting with jaundice and haematemesis on a background of excessive alcohol intake, they would likely be forgiven for listing liver cirrhosis and oesophageal varices at the top of their differentials list [1]. In this article, we present a case involving the above presenting features, but with a subsequent different diagnosis altogether - metastatic malignant melanoma with a gastric metastasis.

This case emphasises the importance of a broad differentials list; the presentation of jaundice and haematemesis in a patient with a history of heavy alcohol use did not lead to a diagnosis of alcohol-related liver disease. In addition, the late diagnosis emphasises the value of initial prevention and early detection of the disease as a mainstay of treatment. Early open disclosure regarding health outcomes and goals of treatment remains important regardless of the timeliness of diagnosis.

## Case presentation

An 87-year-old Caucasian man presented to the emergency department with marked jaundice of two weeks duration and two episodes of haematemesis. The patient estimated both haematemesis episodes to be in the range of 100-200 mL of frank blood, and he continued to experience nausea in the emergency department. He acknowledged having new-onset night sweats in the week leading up to his presentation. In the preceding months, he had been lethargic, had suffered intermittent easy bruising and melaena, and had experienced considerable unintentional weight loss in the context of unexplained anorexia.

His medical history included gout, hyperlipidaemia, and osteoarthritis. He reported good compliance with his allopurinol, meloxicam, and simvastatin, and had no family history of cancer or liver disease. He was fair skinned and had significant sun exposure throughout his working life; while he had no history of skin cancer, there was no evidence of a formal skin cancer check in his history. He also had an extensive travel history throughout Australasia and South-East Asia as a naval officer and had obtained tattoos in Australia, Indonesia, and Japan. He did not have any other risk factors for viral hepatitis. He disclosed that he was a heavy smoker and alcohol drinker until age 45.

At presentation, his vital signs were unremarkable, and examination revealed a large nodular liver extending 4-5 fingerbreadths below the costal margin, a negative murphy’s sign, an ascitic and entirely non-tender abdomen, and extensive jaundice over the praecordium, eyes, and face. A bedside ultrasound suggested that liver metastases and a thickened gallbladder wall were present.

Investigation

His lab results showed elevated liver enzymes with proportionally higher elevations of gamma-glutamyl transferase (GGT) and alkaline phosphatase (ALP) at 443 U/L (range 0-60) and 376 U/L (range 30-110), respectively, indicating pathology of the hepatobiliary system. He had decreased albumin levels at 22 g/L (range 35-52), as well as extended clotting times with an international normalized ratio (INR) of 2.0 (normal range 0.9-1.1), indicating a deficiency in the synthetic function of his liver. Serology was negative for the hepatitis B and hepatitis C viruses, with no evidence of current or past infection, or immunity.

**Figure 1:**
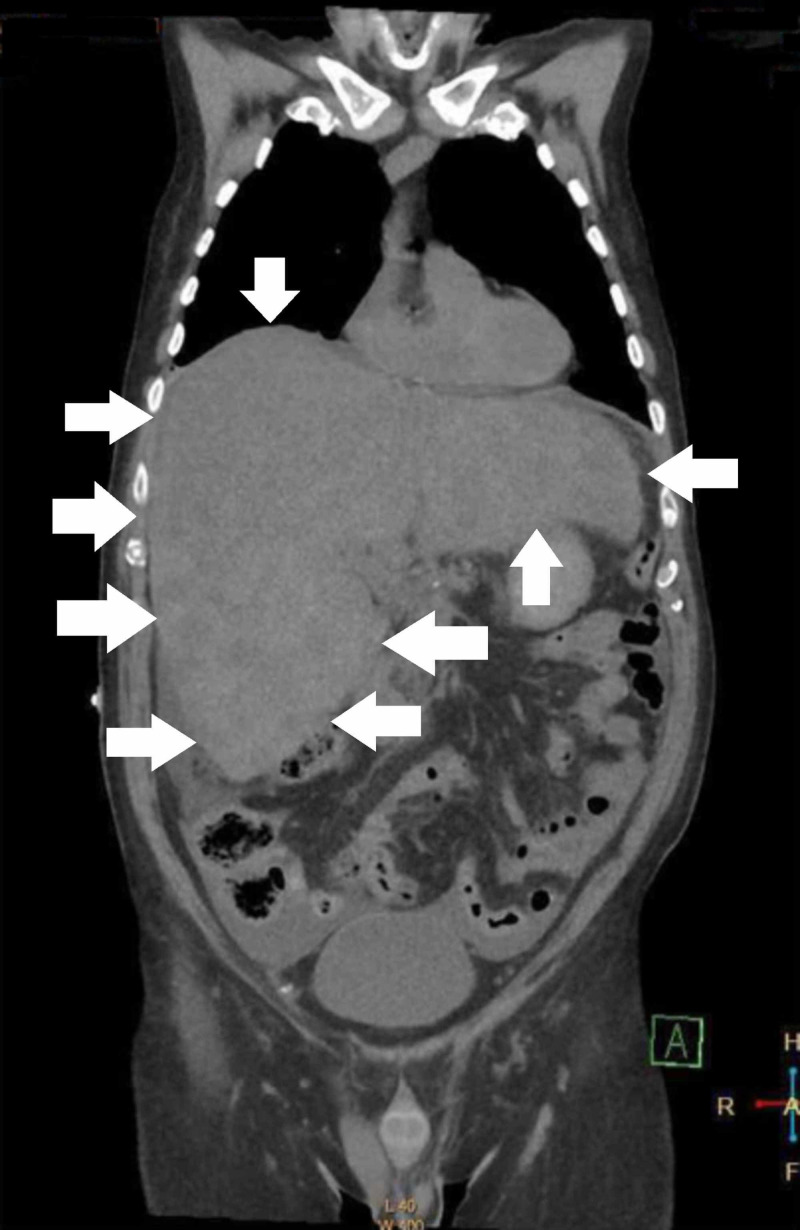
CT of the chest, abdomen, and pelvis, showing a coronal view of the grossly enlarged liver

A chest-abdo-pelvis computed tomography (CT) scan identified a diffusely enlarged liver with multiple low-density nodules, suggestive of either a cirrhotic liver with regenerative nodules or diffuse malignancy; no other possible metastases were identified (Figure 1).

**Figure 2:**
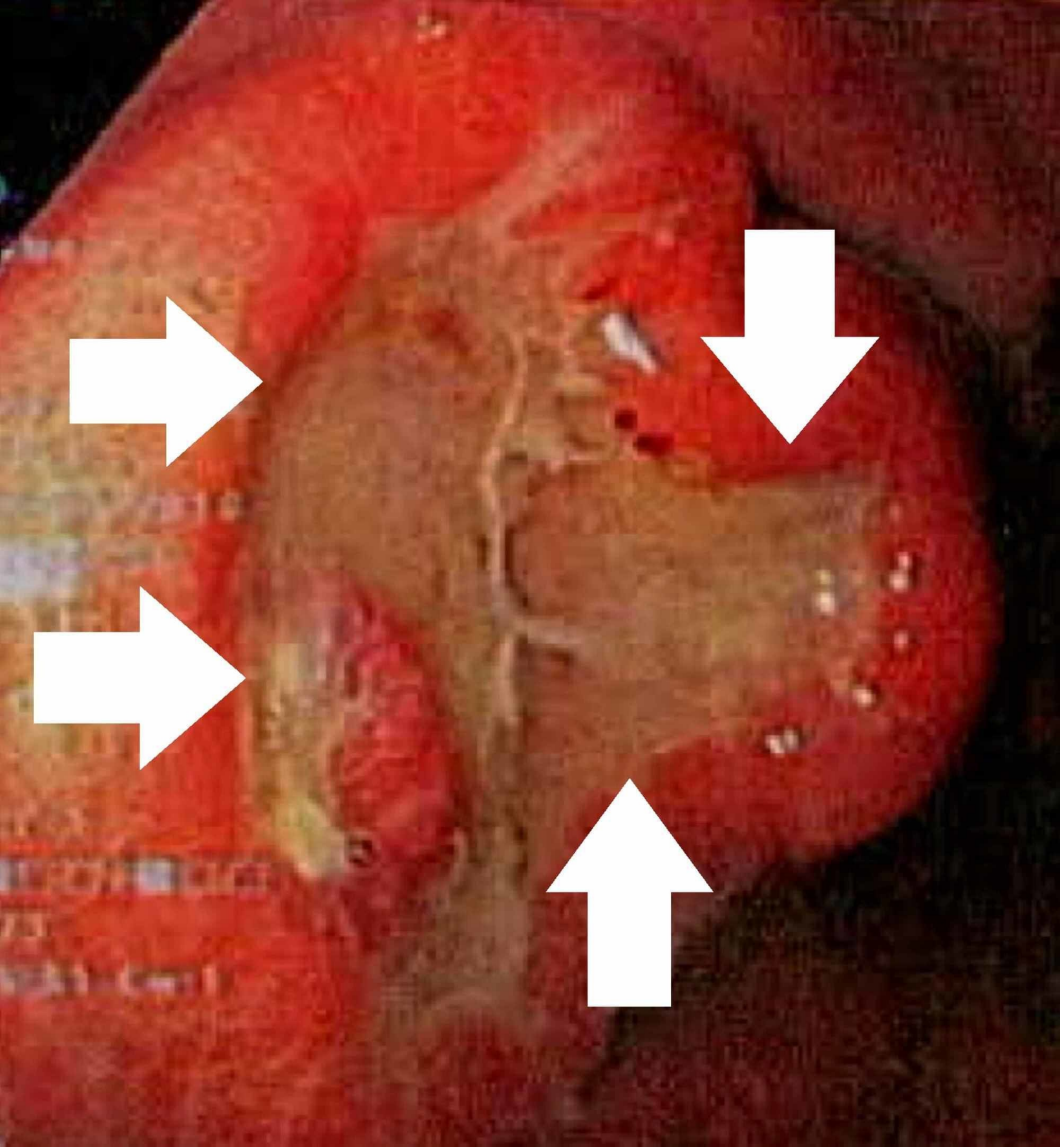
Upper endoscopy photo of the gastric ulcer, depicting the 25 mm lesion with rolled edges and central ulceration – suggestive of metastatic rather than primary origin

A gastroscopy revealed a gastric lesion with rolled edges and central ulceration suggestive of metastatic origin, which was initially histologically diagnosed as an undifferentiated malignant tumour; notably, no oesophageal varices were present (Figure 2).

**Figure 3:**
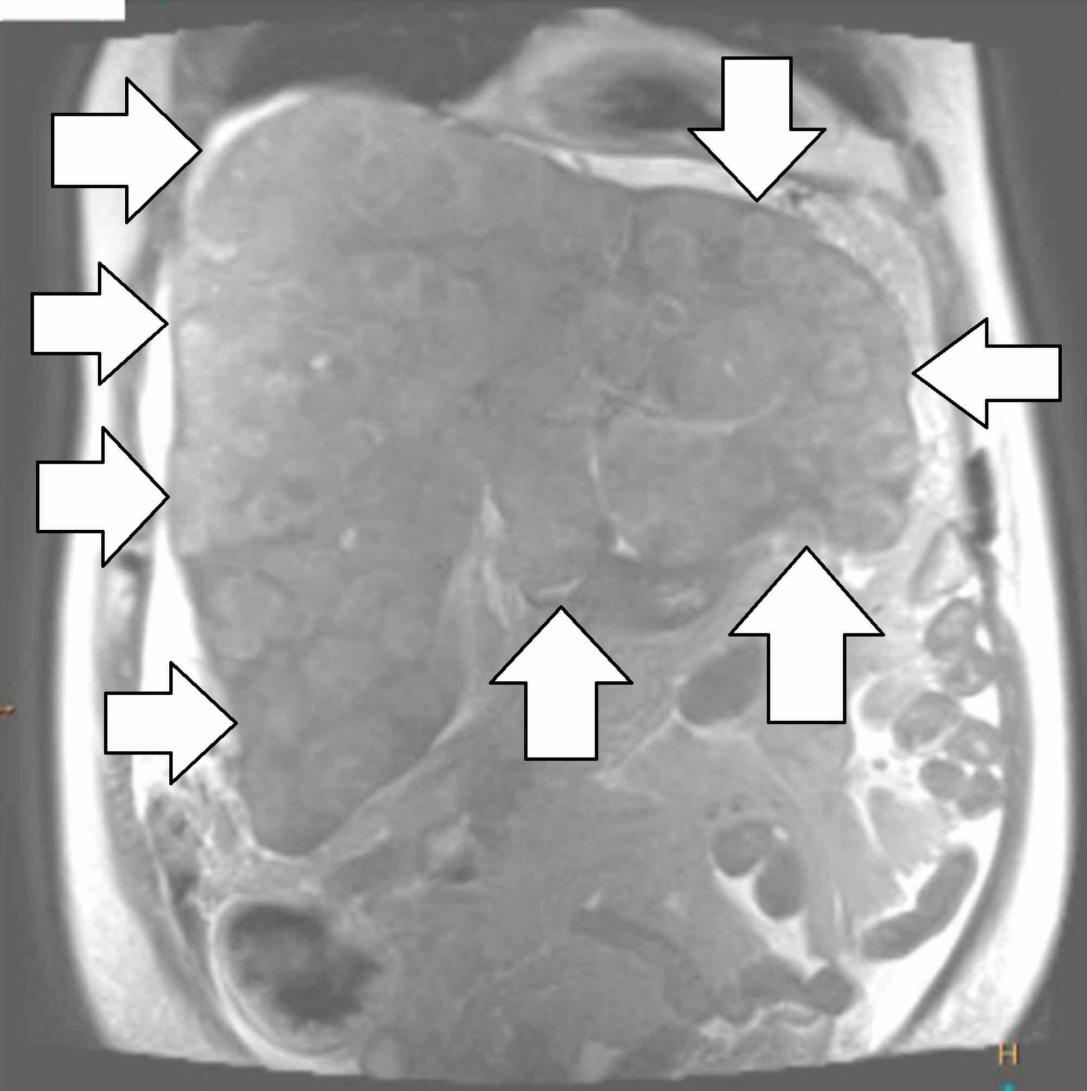
MRCP showing a coronal view of the liver, depicting the innumerable liver metastases MRCP: magnetic resonance cholangiopancreatography.

A magnetic resonance cholangiopancreatography scan again found innumerable hepatic lesions and ruled out cirrhosis due to the irregularity of the liver margins (Figure 3).

**Figure 4:**
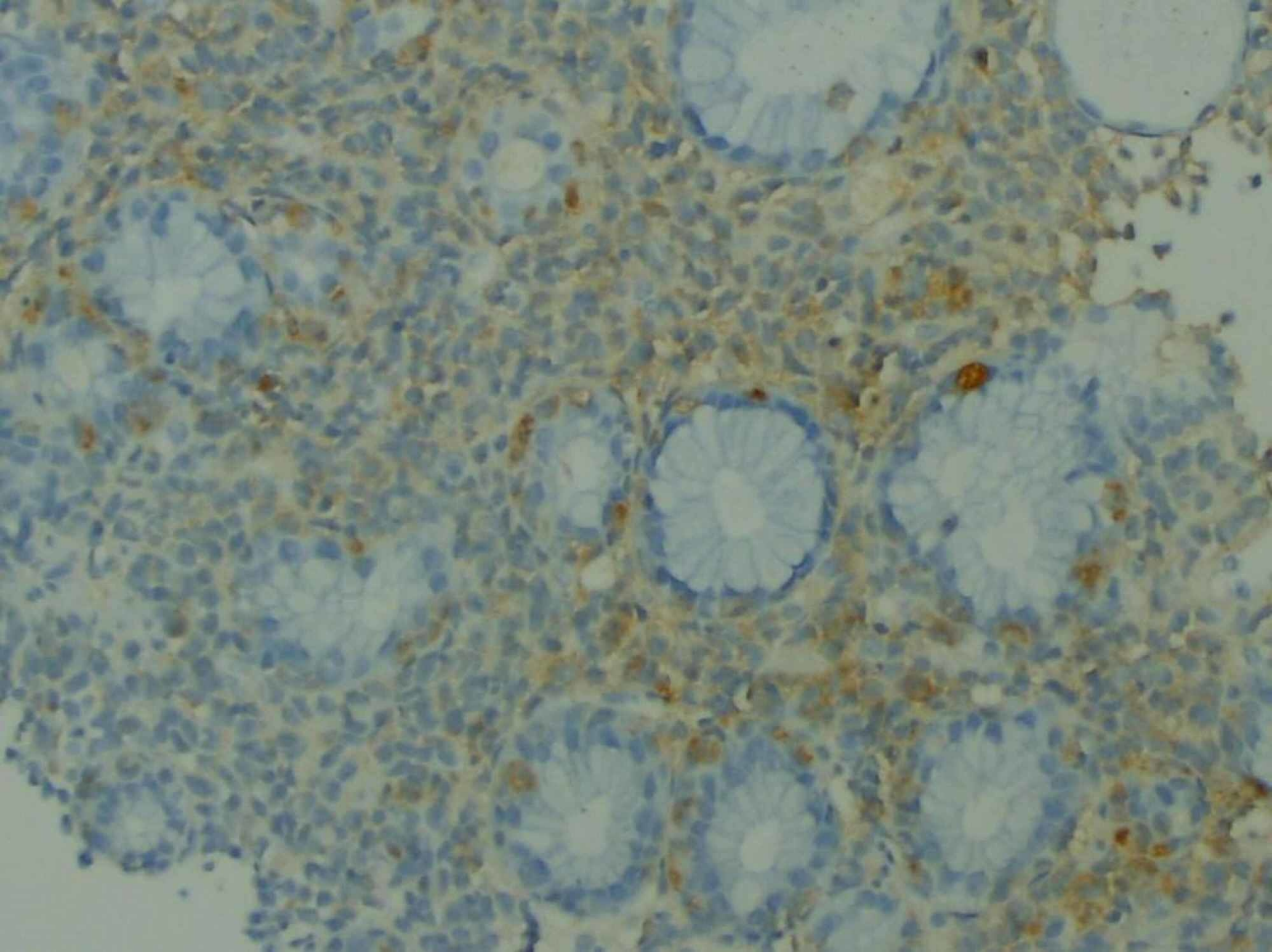
An immunoperoxidase stain showing the infiltrate of malignant cells is positive for S100

**Figure 5:**
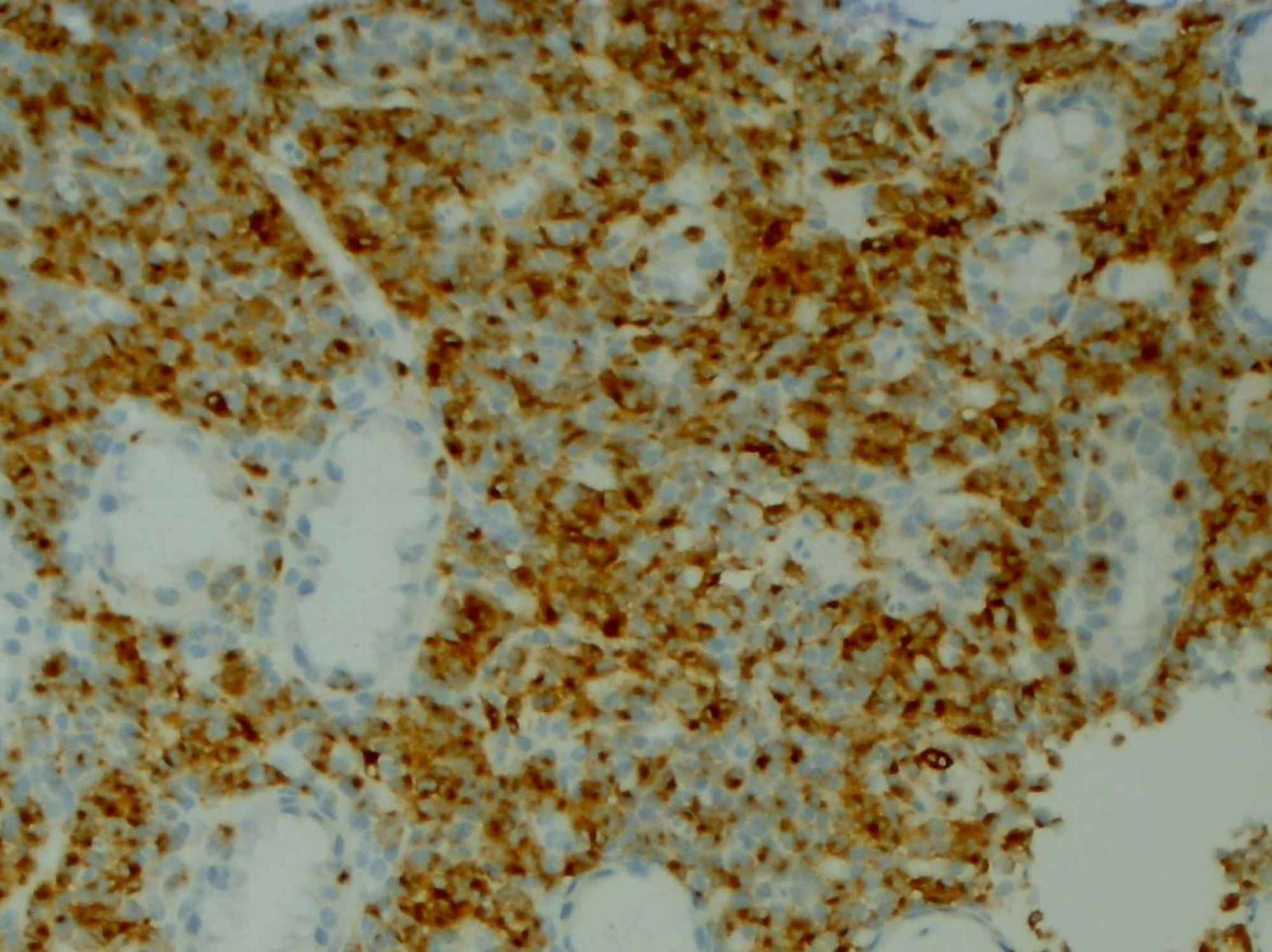
An immunoperoxidase stain showing the infiltrate of malignant cells is positive for Melan-A

On day eight of admission, an immunoperoxidase stain of the gastric biopsy returned as positive for S100 and Melan-A, an immunophenotype that led to the diagnosis of metastatic malignant melanoma (Figures 4-5). No genetic testing was conducted due to the delay in diagnosis.

Outcome and follow up

Although the patient was previously completely independent in activities of daily living, he became progressively drowsier throughout his admission, exhibiting an altered level of consciousness. This was likely due to liver failure. Interventions such as an endoscopic retrograde cholangio-pancreatography and biliary stenting were not considered due to the patient having a hepatic rather than post-hepatic obstructive issue. As the patient appeared relatively comfortable although increasingly drowsy, major palliative interventions were not deemed necessary. He subsequently passed away on day eight of admission.

## Discussion

The reason for the patient’s combined jaundice and haematemesis presentation was unusual, in that it was unrelated to cirrhosis or oesophageal varices despite his history of heavy alcohol use years before. Whilst alcoholic cirrhosis is the most likely cause of jaundice in the elderly, it is often difficult to differentiate liver cancer from cirrhosis upon initial presentation; although jaundice and hepatomegaly are common findings at first presentation of liver cancer, haematemesis is not [1-3]. One case discussed a patient who had gastric metastasis in hepatocellular carcinoma (HCC) causing haematemesis, but only after a documented five-year history of HCC [4].

The patient was noted to have a Type 3 skin type on the Fitzpatrick scale, which is skin that tans uniformly and sometimes burns; however, no obvious skin lesions were noted, and neither a formal skin cancer check nor ophthalmological exam were performed as the diagnosis of a metastatic melanoma only became available on the day of death [5].

One large study reviewed 84,836 patients with melanoma, and found that 91.2%, 5.2%, and 1.3% of the melanomas were cutaneous, ocular, or mucosal in origin, respectively; only 2.2% were of unknown primary type [6]. Further review of the literature suggests that only a very small percentage of melanoma cases involve both the liver and gastrointestinal tract. Of the few reported cases that involved melanoma metastases to the liver and stomach, none had an initial presentation of jaundice and haematemesis [7-14]. Typical presentations of primary or secondary gastric melanoma involved nausea, haematemesis, and abdominal pain, including perhaps non-specific symptoms of fatigue [7-10,11-13]. The above patients typically died within weeks to months of their diagnosis of melanoma.

Metastatic melanoma has historically been a highly fatal condition with a five-year survival rate of 10% [14]. However, the outlook for melanoma has significantly changed in recent years with the advent of targeted mutation immune therapy for melanoma [15]. Had this patient’s symptoms began earlier - leading to an earlier presentation - it is possible he would have been a candidate for this therapy. Where this is not possible, early open disclosure for end of life planning should be considered.

## Conclusions

It is important to keep a broad differentials list when assessing patients for causes of either jaundice or haematemesis, with alcohol-related liver disease initially being the provisional diagnosis. Subsequent investigations, imaging, and pathology results ultimately indicated that metastatic malignant melanoma was the underlying cause of the patient's symptoms. In the context of this patient's deterioration following presentation, early open disclosure of his poor or unclear prognosis enabled the family to say their goodbyes.
